# Sampling Trajectories for the Short-Time Fourier Transform

**DOI:** 10.1007/s00041-022-09977-9

**Published:** 2022-10-19

**Authors:** Michael Speckbacher

**Affiliations:** Faculty of Mathematics, University of Vienna, Oskar-Morgenstern-Platz 1, 1090 Vienna, Austria

**Keywords:** Gabor analysis, Sampling theory, Mobile sampling, Polyanalytic functions, 42C15, 42C30, 42C40, 65T60, 94A20

## Abstract

We study the problem of stable reconstruction of the short-time Fourier transform from samples taken from trajectories in ${\mathbb {R}}^2$. We first investigate the interplay between relative density of the trajectory and the reconstruction property. Later, we consider spiraling curves, a special class of trajectories, and connect sampling and uniqueness properties of these sets. Moreover, we show that for window functions given by a linear combination of Hermite functions, it is indeed possible to stably reconstruct from samples on some particular natural choices of spiraling curves.

## Introduction

Reconstructing a function from incomplete data is an omnipresent task in fields like signal processing or harmonic analysis. Classically, one aims to reconstruct a function from discrete samples (see e.g. [34, 38]) and the stable version of this problem leads to the notion of frames [12, 15]. For a variety of function spaces, the lower Beurling density [10] (the definition being adapted to the particular geometry of the function space in consideration) of the discrete sampling set needs to exceed a certain threshold in order for the sampling process to form a frame, see, for example, [1, 24, 31, 33]. This threshold is commonly known as the *Nyquist rate*.

A more abstract form of the sampling problem is to characterize so called *sampling measures* [6, 27, 28], i.e. measures $\mu$ on *X* that satisfy

$$\begin{aligned} A\Vert F\Vert _{L^p(X)}^p\le \int _X |F|^p\,\textrm{d}\mu \le B\Vert F\Vert _{L^p(X)}^p, \end{aligned}$$

for some constants $A,B>0$ and every *F* in a given subspace of $L^p(X)$. A common situation is that $X={\mathbb {R}}^d$ and the measure takes the form $\mu =\chi _\varGamma {\mathcal {H}}^k$, where $\varGamma \subset X$, $\chi _\varGamma$ is the characteristic function on $\varGamma$, and ${\mathcal {H}}^k$, $0\le k\le d$, is the *k*-dimensional Hausdorff measure. The case $k=0$ corresponds to the classical discrete sampling problem. For the second extreme case $k=d$, it turns out that a weaker notion of density is often sufficient to stably reconstruct. For the Paley-Wiener space, this is known as the Logvinenko-Sereda theorem [23, 25] and multiple extensions to other spaces of analytic functions [18, 26, 28] and the range space of the short-time Fourier transform [20] have since been established.

Recently, the study of the intermediate cases $0<k<d$ on the Paley-Wiener space $PW_2(\varOmega )$ has drawn increasing attention and became known in the literature as the *mobile sampling problem* [36, 37]. Mobile sampling has many applications whenever a signal is measured by a moving sensor like in an MRI scan [8, 36]. Adapting Beurling’s lower density for $k=1$, the *lower path density* of a set $\varGamma$ (measuring the limit average length of the curve $\varGamma$ in balls of increasing radii) was studied in [17]. In [22], it is shown that a sufficiently large lower path density yields a stable sampling process. On the other hand, there is no Nyquist rate that the lower path density needs to exceed for stable sampling, see [17]. For particularly structured classes of trajectories $\varGamma$ however, Nyquist rates were established in terms of certain parameters that characterize the ’separation’ of $\varGamma$ [21, 32].

In this paper, we study the equivalent of the mobile sampling problem for the *short-time Fourier transform*

$$\begin{aligned} V_gf(z)=\int _{\mathbb {R}}f(t)\overline{g(t-x)}e^{-2\pi i \xi t}\,\textrm{d}t, \quad z=(x,\xi )\in {\mathbb {R}}^2. \end{aligned}$$

The *modulation spaces* $M^p_\vartheta ({\mathbb {R}})$ are defined by

$$\begin{aligned} M^p_v({\mathbb {R}}):=\left\{ f\in {\mathcal {S}}'({\mathbb {R}}):\ V_{h_0}f\cdot \vartheta \in L^p({\mathbb {R}}^2)\right\} , \end{aligned}$$

where $h_0$ denotes the standard Gaussian, and $\vartheta$ is a submultiplicative weight, see, e.g., [15]. We equip $M^p_v({\mathbb {R}})$ with the natural norm $\Vert f\Vert _{M^p_\vartheta }=\Vert V_{h_0}f\cdot \vartheta \Vert _{L^p}$, and write $M^p({\mathbb {R}})=M^p_\vartheta ({\mathbb {R}})$ if $\vartheta \equiv 1$. This article is concerned with the study of *Gabor sampling trajectories* for $M^p({\mathbb {R}})$, that is, trajectory sets $\varGamma \subset {\mathbb {R}}^2$ for which

1 $$\begin{aligned} A\Vert f\Vert ^p_{M^p}\le \int _\varGamma |V_g f(z)|^p\,\textrm{d}{\mathcal {H}}^1(z)\le B\Vert f\Vert ^p_{M^p},\quad f\in M^p({\mathbb {R}}), \end{aligned}$$

holds. This is a particular instance of a sampling measure for the short-time Fourier transform [6].

Just like in classical Gabor analysis, the stable sampling property relies heavily on both the window *g* and the trajectory set $\varGamma$. On the one hand, one can quickly show that for windows in $M^1({\mathbb {R}})$, a necessary condition for $\varGamma$ being a Gabor sampling trajectory is that it is *relatively dense* [6] (see Sect. 3.1 for the definition of relative density). On the other hand, applying a characterization of sampling measures on the Bargmann-Fock space [28] we show that relative density is also sufficient for Gabor sampling trajectories with Gaussian window (see Sect. 3.2). It is thus hopeless to search for a Nyqist rate (both in terms of a path density as well as certain separation parameters) that needs to be exceeded for general windows. However, in Proposition 4 we prove that for certain windows a sufficient condition for $\varGamma$ being a Gabor sampling trajectory is that, for $R\le R_g$ and every $z\in {\mathbb {R}}^2$, ${\mathcal {H}}^1(\varGamma \cap B_R(z))$ is bounded away from zero.

**Fig. 1:**
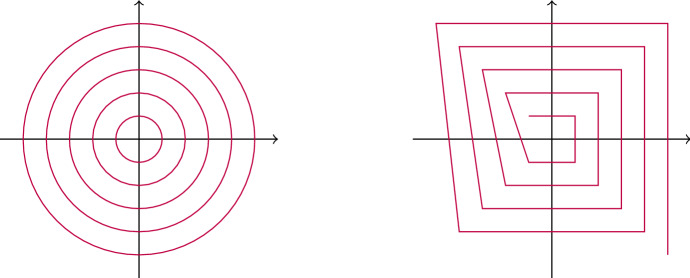
Two examples of spiraling curves. *Left* The set of concentric circles $O_\eta$. *Right* The path ${\mathcal {S}}_\eta (z_1,\ldots ,z_4)$ generated by the points $\{z_1,\ldots ,z_4\}=\{(-a,a),(a,a),(a,-a),(-a,-a)\}$, $a>0$

Later, we change perspective and study particular examples of trajectory sets. First, we characterize sampling and uniqueness properties if $\varGamma$ is a collection of parallel lines. This will in turn be useful when studying the sampling property of a particular class of trajectories: *spiraling curves*. Spiraling curves were introduced in [21] where their sampling properties on the Paley-Wiener space were studied. In this article, we work with a slightly more restrictive definition of spiraling curves that nevertheless includes all the examples given in [21]. Loosely speaking, a spiraling curve is a trajectory set that, when shifted in a particular direction, approaches a collection of equispaced parallel lines or equispaced parallel edges in the limit. We spare the technical details for now and refer to Definition 3 and Theorem 4 (Fig. 1).

Beurling’s theory of weak limits [10, 11, 34] establishes an equivalence between sampling sets $\varGamma$ for the Paley-Wiener space $PW_2(\varOmega )$ and the uniqueness property for all weak limits of translates of $\varGamma$ on the Bernstein space $PW_\infty (\varOmega )$. This is a very powerful result as the uniqueness property is often easier to verify. The price one has to pay is that uniqueness has to be shown on a larger space and for all weak limits of translates of the original sampling set. A similar result for sampling measures for the modulation space $M^p({\mathbb {R}})$ was shown by Ascensi [6]. In case of the measure $\chi _\varGamma \,\textrm{d}{\mathcal {H}}^1$, this result states that $\varGamma$ is a Gabor sampling trajectory for $M^p({\mathbb {R}})$ if and only if all weak limits of translates of $\varGamma$ are uniqueness sets for the weighted modulation space $M^p_{1/\vartheta _s}({\mathbb {R}})$, for some $s>2$ and $\vartheta _s(z)=(1+|z|)^s$.

In most applications, Beurling’s theory is used to derive necessary conditions on sampling sets. In [21], for example, the authors showed that certain weak limits of translates of spiraling curves cannot be uniqueness sets for $PW_\infty (\varOmega )$ if the separation exceeds a given threshold. Together with a result from [8], this yields a full characterization which sets of concentric circles and which Archimedes spirals are sampling trajectories in terms of their separation parameter [21, Theorem A].

We take a different approach in this paper in that we fully characterize the set of weak limits of translates of spiraling curves and then show (Theorem 4) that, for windows taken from the linear span of the Hermite functions, the uniqueness property is automatically satisfied for all weak limits of $\varGamma$ except for $\varGamma$ itself (and its finite shifts). It then follows that $\varGamma$ is a Gabor sampling trajectory for $M^p({\mathbb {R}})$ if and only if $\varGamma$ is a uniqueness set for $M^p_{1/\vartheta _s}({\mathbb {R}})$, for some $s>2$ (Theorem 5). Moreover, we prove that certain spiraling curves are indeed such uniqueness sets. In particular, our main result is the following.

### Theorem 1

Let $h_n,\ n\in {\mathbb {N}}_0$, denote the Hermite functions (defined in (5)), $g\in \text {span}\{h_n: n\in {\mathbb {N}}_0\}$, $1\le p<\infty$, and $\eta >0$. Moreover, let

- *P* be a star shaped polygon in ${\mathbb {R}}^2$ with kernel *K* such that the origin is contained in the interior of *K*,
- $z_1,\ldots ,z_N \in {\mathbb {R}}^2,\ N\ge 3$, be such that $\text {arg}(z_1),\ldots ,\text {arg}(z_N),\text {arg}(z_1 )$ is strictly increasing or strictly decreasing modulo $2\pi$.

The following sets are Gabor sampling trajectories for $M^p({\mathbb {R}})$:

(i) the set of concentric circles ${\mathcal {O}}_\eta :=\{(x,y)\in {\mathbb {R}}^2 :\ x^2+y^2=\eta ^2k^2,\ k\in {\mathbb {N}}\},$
(ii) the collection of star shaped polygons ${\mathcal {P}}_\eta :=\{(x,y)\in {\mathbb {R}}^2:\ (x,y)\in \eta k P,\ k\in {\mathbb {N}}\},$
(iii) ${\mathcal {S}}_\eta (z_1,...,z_N)$, the path generated by the sequence of vectors
  $$\begin{aligned} \{\eta z_1,\eta z_2,...,\eta z_N,2\eta z_1,2\eta z_2,...,2\eta z_N,3\eta z_1,3\eta z_2,...\}. \end{aligned}$$

This paper is organized as follows. After a section dedicated to preliminaries, we study some basic examples of windows and trajectory sets and give necessary and sufficient conditions for Gabor sampling trajectories in that setting in Sect. 3. Then in Sect. 4 we introduce spiraling curves and study their weak limits of translates which then allows us to prove our main result.

## Preliminaries

### Notation

Throughout this paper we adopt the following conventions. We write $\vec {d}$ for vectors in ${\mathbb {S}}^1$ and $\vec {\ell }(\theta ):=(\cos (2\pi \theta ),\sin (2\pi \theta ))$, $\theta \in {\mathbb {T}}\cong [0,1)$ as well as $\vec {e}_i,\ i=1,2,$ for the standard basis vectors in ${\mathbb {R}}^2$. For $\vec {d}\in {\mathbb {S}}^1$ we write $\vec {d}_\bot$ for the vector obtained by rotating $\vec {d}$ clockwise by $\pi /2$. The space of continuous, compactly supported functions is denoted by $C_c({\mathbb {R}}^d)$, the ball of radius *R* and center *z* by $B_R(z)$, and we write $A\lesssim B$ if there exist $C>0$ such that $A\le CB$.

### Hausdorff Measures and Trajectory Sets

Let $E\subset {\mathbb {R}}^2$. The *1-dimensional Hausdorff measure* of *E* is given by

$$\begin{aligned} {\mathcal {H}}^1(E)=\lim _{\delta \searrow 0}\inf \left\{ 2\sum _j r_j:\ E\subset \bigcup _j B(x_j,r_j)\ \text {and}\ r_j\le \delta \right\} . \end{aligned}$$

When restricted to a line, ${\mathcal {H}}^1$ equals the 1-dimensional Lebesgue measure.

Let $\varphi :[0,1)\rightarrow [0,\infty )$ be continuous at 0. A (locally finite Borel) measure $\mu$ on ${\mathbb {R}}^2$ is called $\varphi$*-regular* if for every $z\in {\mathbb {R}}^2$ and every $R\in (0,1)$ one has

$$\begin{aligned} \mu (B_R(z))\le \pi \varphi (R) R. \end{aligned}$$

A set $E\subset {\mathbb {R}}^2$ is called $\varphi$*-regular* if ${\mathcal {H}}^{1}|_E$ is $\varphi$-regular.

A *trajectory* $\varGamma \subset {\mathbb {R}}^d$ is the image of a curve $\gamma :{\mathbb {R}}\rightarrow {\mathbb {R}}^d$, i.e. $\varGamma =\gamma ({\mathbb {R}})$, such that each restriction of $\gamma$ to a finite interval is rectifiable. A *trajectory set* $\varGamma$ is a countable collection of trajectories.

### The Short-Time Fourier Transform and Modulation Spaces

Let $z=(x,\xi )\in {\mathbb {R}}^2$. A *time-frequency shift* of a function *g* is given by

$$\begin{aligned} \pi (z)g(t)=M_\xi T_x g(t)=e^{2\pi i\xi t}g(t-x). \end{aligned}$$

Given a window $g\in L^2({\mathbb {R}})$, the *short-time Fourier transform* of $f\in L^2({\mathbb {R}})$ is defined as

$$\begin{aligned} V_g f(z)=\langle f,\pi (z)g\rangle =\int _{\mathbb {R}}f(t)\overline{g(t-x)}e^{-2\pi i \xi t}\,\textrm{d}t. \end{aligned}$$

The short-time Fourier transform satisfies the following *orthogonality relation*

$$\begin{aligned} \int _{{\mathbb {R}}^2}V_{g_1}f_1(z)\overline{V_{g_2}f_2(z)}\,\textrm{d}z=\langle f_1,f_2\rangle \overline{\langle g_1,g_2\rangle }, \end{aligned}$$

which implies that, if $\Vert g\Vert _2=1$, $V_g:L^2({\mathbb {R}})\rightarrow L^2({\mathbb {R}}^2)$ is an isometry.

Let $\vartheta :{\mathbb {R}}^2\rightarrow {\mathbb {R}}^+$ be a *submultiplicative weight function*, i.e. $\vartheta (z+w)\le \vartheta (z)\vartheta (w)$. The *(weighted) modulation spaces* $M_{\vartheta }^p({\mathbb {R}})$, $1\le p\le \infty$, can be defined by

$$\begin{aligned} M_{\vartheta }^p({\mathbb {R}}):=\big \{f\in {\mathcal {S}}'({\mathbb {R}}):\ V_{h_0}f \cdot \vartheta \in L^p({\mathbb {R}})\big \}, \end{aligned}$$

where $h_0$ denotes the normalized Gaussian and ${\mathcal {S}}'({\mathbb {R}})$ the space of tempered distributions. Modulation spaces are Banach spaces when equipped with the natural norm $\Vert f\Vert _{M_\vartheta ^p} =\Vert V_{h_0}f\cdot \vartheta \Vert _{L^p}$. If $\vartheta \equiv 1$, we write $M^p({\mathbb {R}})=M_\vartheta ^p({\mathbb {R}})$. See, for example, [9, 15] for a thorough introduction to the topic. Throughout this paper we only consider *polynomial weight functions*, i.e.,

$$\begin{aligned} \vartheta _s(z)=(1+|z|)^s,\quad z\in {\mathbb {R}}^2,\ s\ge 0. \end{aligned}$$

We note here that $M_{\vartheta _s}^1({\mathbb {R}})$ is closed under pointwise multiplication with functions from the *weighted Fourier algebra* $A_{\vartheta _s}({\mathbb {R}})$

$$\begin{aligned} A_{\vartheta _s}({\mathbb {R}}):=\left\{ f\in C_0({\mathbb {R}}):\ \int _{\mathbb {R}}|{\widehat{f}}(\xi )|\vartheta _s(\xi )\,\textrm{d}\xi <\infty \right\} . \end{aligned}$$

In particular, for $f\in A_{\vartheta _s}({\mathbb {R}})$ and $g\in M_{\vartheta _s}^1({\mathbb {R}})$ we have

2 $$\begin{aligned} \Vert fg\Vert _{M_{\vartheta _s}^1 }\le \Vert f\Vert _{A_{\vartheta _s}}\Vert g\Vert _{M_{\vartheta _s}^1 }, \end{aligned}$$

see, for example, the arguments in [19, Proposition 4.13] which can easily be adapted to the weighted case.

Let $g\in M^1({\mathbb {R}})$. We call a trajectory set $\varGamma \subset {\mathbb {R}}^2$ a *Gabor sampling trajectory* for $M^p({\mathbb {R}})$ if there exist constants $A,B>0$ such that

3 $$\begin{aligned} A\Vert f\Vert ^p_{M^p}\le \int _\varGamma |V_g f(z)|^p\,\textrm{d}{\mathcal {H}}^1(z)\le B\Vert f\Vert ^p_{M^p},\quad f\in M^p({\mathbb {R}}). \end{aligned}$$

If only the upper bound is satisfied, then we call $\varGamma$ a *Gabor Bessel trajectory*. If $p=2$, then $M^p({\mathbb {R}})=L^2({\mathbb {R}})$, and (3) is equivalent to $\{\pi (z)g\}_{z\in \varGamma }$ forming a continuous frame, see [5, 30].

The trajectory set $\varGamma$ is called a *uniqueness set* for $M^p({\mathbb {R}})$, if $V_gf|_\varGamma =0$, $f\in M^p({\mathbb {R}})$, implies $f=0$.

### Hermite Windows and Polyanalytic Functions

A function $F:{\mathbb {C}}\rightarrow {\mathbb {C}}$ is called *polyanalytic of order n* if it satisfies the higher order Cauchy-Riemann equation $({\bar{\partial }})^{n+1}F=0$. In that case, *F* can be written as

4 $$\begin{aligned} F(z)=F(z,{\overline{z}})=\sum _{k=0}^n F_k(z){\overline{z}}^k,\quad z\in {\mathbb {C}}, \end{aligned}$$

where $F_0,\ldots ,F_n:{\mathbb {C}}\rightarrow {\mathbb {C}}$ are holomorphic functions.

The *Hermite functions* are given by

5 $$\begin{aligned} h_n(t)=\frac{2^{1/4}}{\sqrt{n!}}\left( \frac{-1}{2\sqrt{\pi }}\right) ^n e^{\pi t^2}\frac{d^n}{dt^n}(e^{-2\pi t^2}),\quad n\in {\mathbb {N}}_0. \end{aligned}$$

For $f\in M^p_{\vartheta _s}({\mathbb {R}})$, $1\le p<\infty$, the function *F* given by

6 $$\begin{aligned} F(z)=V_{h_n}f({\overline{z}})e^{\pi (z^2-{\overline{z}}^2)/4} e^{\pi |z|^2/2}, \end{aligned}$$

is polyanalytic of order *n* [2], where we identify $z=(x,\xi )\in {\mathbb {R}}^2$ with $z=x+i\xi \in {\mathbb {C}}$. In particular, if $g=\sum _{k=0}^n \alpha _k h_k$, then $V_{g}f({\overline{z}})e^{\pi (z^2-{\overline{z}}^2)/4} e^{\pi |z|^2/2}$ is polyanalytic of order *n*.

A polyanalytic function *F* of order *n* is called *reduced* if it can be written as

$$\begin{aligned} F(z)=\sum _{k=0}^n F_k(z)|z|^{2k},\quad F_k\text { holomorphic.} \end{aligned}$$

Reduced polyanalytic functions satisfy the following Cauchy-type formula [7, Sect. 1.3, (11)]. For a similar Cauchy-type formula for true polyanalytic functions, we refer to [4].

#### Lemma 1

(Balk) Let *F* be a reduced polyanalytic function of order *n* in $B_R(0)$, $0<R_0<R_1<\ldots<R_n<R$, and let $\varGamma _k:=\{z:\ |z|=R_k\}$. For every $z\in B_{R_0}(0)$

7 $$\begin{aligned} F(z)=\frac{1}{2\pi i}\sum _{k=0}^n P_k(|z|^2)\int _{\varGamma _k}\frac{F(t)}{t-z}dt, \end{aligned}$$

where $$ P_k(t):=\prod _{j\ne k}\frac{R_j^2-t}{R_j^2-R_k^2}. $$

### The Metaplectic Rotation

Let us denote the rotation matrices in ${\mathbb {R}}^2$ by $$ R(\theta )=\left( {\begin{matrix} \cos ( 2\pi \theta ) &{} -\sin (2\pi \theta )\\ \sin (2\pi \theta ) &{}\cos (2\pi \theta ) \end{matrix}}\right) ,\ \theta \in {\mathbb {T}}. $$ The *metaplectic rotation* of $f\in L^2({\mathbb {R}})$ is given in terms of the Hermite basis $\{h_n\}_{n\in {\mathbb {N}}_0}$

$$\begin{aligned} U(\theta ) f:=\sum _{n\ge 0}e^{-2\pi in\theta }\langle f,h_n\rangle h_n. \end{aligned}$$

Clearly, $U(\theta )$ is a unitary operator on $L^2({\mathbb {R}})$ with $U(\theta )^*=U(-\theta )$, and $U(\theta ) h_n=e^{-2\pi in\theta } h_n$. For $f,g\in L^2({\mathbb {R}})$, the standard rotation of the argument of the short-time Fourier transform and the metaplectic rotation are connected via the formula

8 $$\begin{aligned} V_gf (R(\theta ) z)=e^{\pi i (x\omega -x'\omega ')}V_{U({ \theta }) g}U({ \theta })f(z),\quad z=(x,\omega ),\text { and }(x'\omega ')=R(\theta ) z,\nonumber \\ \end{aligned}$$

or equivalently via $\pi (R(\theta ) z)=e^{-\pi i (x\omega -x'\omega ')}U(-\theta )\pi (z)U(\theta )$. This is a special case of the symplectic covariance of the Schrödinger representation, see [13, Chaps. 1 and 2], and [15, Chap. 9].

As $\vartheta _s$ is radially symmetric, (8) implies that $\Vert f\Vert _{M^1_{\vartheta _s} }=\Vert U(\theta )f\Vert _{M^1_{\vartheta _s} }$, which allows to extend the operator $U(\theta )$ to $M^\infty _{1/\vartheta _s}({\mathbb {R}})=M^1_{\vartheta _s}({\mathbb {R}})^*$ via

$$\begin{aligned} \langle U(\theta )f,g\rangle _{M^\infty _{1/\vartheta _s}\times M^1_{\vartheta _s}}=\langle f,U(-\theta )g\rangle _{M^\infty _{1/\vartheta _s}\times M^1_{\vartheta _s}},\quad f\in M^\infty _{1/\vartheta _s}({\mathbb {R}}),\ g\in M^1_{\vartheta _s}({\mathbb {R}}). \end{aligned}$$

Consequently, (8) remains valid for $f\in M^\infty _{1/\vartheta _s}({\mathbb {R}}),$ and $g\in M^1_{\vartheta _s}({\mathbb {R}})$.

## General Trajectory Sets

In this section, we present some basic necessary and sufficient conditions for Gabor sampling trajectories. Moreover, we study the cases of (i) sampling on general trajectory sets for specific windows, in particular, the Gaussian window (Corollary 1) and a certain class of window functions (Proposition 4), and (ii) sampling on parallel lines for general windows (Proposition 2).

### Relative Density

We call a trajectory set $\varGamma \subset {\mathbb {R}}^2$ *(m, R)-dense* if

$$\begin{aligned} \inf _{z\in {\mathbb {R}}^2}{\mathcal {H}}^1(\varGamma \cap B_R(z) )\ge m>0, \end{aligned}$$

and *relatively dense* if there exist constants $m,R>0$ such that $\varGamma$ is (*m*, *R*)-dense. It turns out that relative density is a necessary condition for $\varGamma$ being a Gabor sampling trajectory for $M^p({\mathbb {R}})$, see [6, Theorems 8 and 10]:

#### Proposition 1

(Ascensi) Let $g\in M^1({\mathbb {R}})$ and $1\le p<\infty$. If $\varGamma \subset {\mathbb {R}}^2$ is a Gabor Bessel trajectory for $M^p({\mathbb {R}})$, then there exist $M,R>0$ such that

9 $$\begin{aligned} \sup _{z\in {\mathbb {R}}^2}{\mathcal {H}}^1(\varGamma \cap B_{R}(z))\le M. \end{aligned}$$

If $\varGamma$ is a Gabor sampling trajecory for $M^p({\mathbb {R}})$, then $\varGamma$ is relatively dense.

Note that the proof of (9) can easily be adapted to the case $p=2$ and general $g\in L^2({\mathbb {R}})$ and explicit upper bounds of the Bessel constant can be derived, for example, from [3, 4].

The question whether relative density is also necessary for general windows and Gabor sampling trajectories for $L^2({\mathbb {R}})$ remains open. Drawing comparison to the discrete [31] and planar cases [20] however suggests that relative density should indeed be a necessary requirement.

### Gaussian Window

In [28], Ortega-Cerdà fully characterized the sampling measures for the Bargmann-Fock space of entire functions which corresponds to the short-time Fourier transform with Gaussian window $h_0$. The result goes as follows.

#### Theorem 2

(Ortega-Cerdà) Let $1\le p<\infty$. The measure $\mu$ is a sampling measure for $V_{h_0}(M^p({\mathbb {R}}))$ if and only if there exist constants $R,\delta ,M>0$ and $N\in {\mathbb {N}}$ such that

$$\begin{aligned} (i)\ \sup _{z\in {\mathbb {R}}^2}\mu (B_R(z))\le M,\quad \quad \quad (ii)\ \inf _{z\in {\mathbb {R}}^2}\frac{n(R,N,\delta ,z)}{R^2}>1, \end{aligned}$$

where $n(R,N,\delta ,z)$ is calculated by the following rule: take $S_R(z):=z+[-R/2,R/2)^2$ and cover it with $N^2$ smaller squares of sidelength *R*/*N*. Then $n(R,N,\delta ,z)$ denotes the number smaller squares $s\subset S_R(z)$ that satisfy $\mu (s)\ge \delta$.

When specifying the measure $\mu$ to be the 1-dimensional Hausdorff measure on $\varGamma$, we subsequently show that, with a minor extra condition on the trajectory set $\varGamma$, condition (ii) is equivalent to $\varGamma$ being relatively dense.

#### Corollary 1

Let $g=h_0$ be the standard Gaussian window, $1\le p<\infty ,$ $\varphi :[0,1]\rightarrow [0,\infty )$ be continuous at 0, and $\varGamma \subset {\mathbb {R}}^2$ be a $\varphi$-regular trajectory set. Then $\varGamma$ is a Gabor sampling trajectory for $M^p({\mathbb {R}})$ if and only if there exist $R,m,M>0$ such that

10 $$\begin{aligned} m\le {\mathcal {H}}^1(\varGamma \cap B_R(z))\le M,\quad \text {for every }z\in {\mathbb {R}}^2. \end{aligned}$$

#### Proof

The second inequality of (10) is condition (i) in Theorem 2. Hence, we have to show the equivalence of the left hand side inequality and (ii). If (ii) holds, then

$$\begin{aligned} {\mathcal {H}}^1(\varGamma \cap B_{R}(z))\ge {\mathcal {H}}^1(\varGamma \cap S_R(z))\ge \delta n(R,N,\delta ,z)>\delta R^2. \end{aligned}$$

Now, let $\varGamma$ be relatively dense. Note that we may assume that $\varphi (0)>0$ (for if $\varGamma$ is $\varphi$-regular, then it is also $(\varphi +\varepsilon )$-regular for any $\varepsilon >0$). Let us choose *N* large enough such that $\sqrt{2}R/N<1$, and $\varphi (\sqrt{2}R/N)\le \sqrt{2}\varphi (0)$. Then, using the lower bound in (10) and the $\varphi$-regularity of $\varGamma$, we get

$$\begin{aligned} m&\le {\mathcal {H}}^1(\varGamma \cap B_R(z)) \le {\mathcal {H}}^1(\varGamma \cap S_{2R}(z)) \\&\le n(2R,N,\delta ,z)\sup _{w\in S_{2R}(z)}{\mathcal {H}}^1(\varGamma \cap S_{\frac{2R}{N}}(w)) +(N^2-n(2R,N,\delta ,z))\delta \\&\le n(2R,N,\delta ,z)2\pi \varphi (0)\frac{ R}{N}+(N^2-n(2R,N,\delta ,z))\delta . \end{aligned}$$

Regrouping the terms yields and choosing $\delta =\delta _N=\frac{1}{N^2}\min \{m/2,\pi R\varphi (0)N\}$ shows that

$$\begin{aligned} n(2R,N,\delta ,z))\ge N\frac{m-N^2\delta }{2\pi R\varphi (0)-\delta N}\ge N \frac{m}{4\pi R\varphi (0)}, \end{aligned}$$

which can be arbitrarily large as $N\rightarrow \infty$. In particular, there exists $N\in {\mathbb {N}}$ such that (*ii*) is satisfied. $\square$

#### Remark 1

In [29] it is shown that $\varphi$-regularity with $\varphi (0) =1$ can be considered as a quantitative strengthening of rectifiability. On the other hand, it is possible to construct fractal sets that are $\varphi$-regular if $\varphi (0) > 1$, which shows that $\varphi$-regularity for some $\varphi$ is a rather mild assumption.

### The Case of Parallel Lines

We now study sampling and uniqueness properties of sets of parallel lines which follow from simple arguments. The results however will be useful later when we study spiraling curves.

#### Proposition 2

Let $g\in L^2({\mathbb {R}})$, $\varLambda \subset {\mathbb {R}}$ be countable, and $\vec {d}=R(\theta )\vec {e}_2$. The collection of parallel lines $L_{\vec {d},\varLambda }$, where

$$\begin{aligned} L_{\vec {d},\varLambda }:= \big \{t\vec {d} + \lambda \vec {d}_\bot :\ t\in {\mathbb {R}},\ \lambda \in \varLambda \big \}, \end{aligned}$$

is a Gabor sampling trajectory for $L^2({\mathbb {R}})$ with sampling bounds $A,B>0$ if and only if

11 $$\begin{aligned} A\le \sum _{\lambda \in \varLambda }|U(\theta ) {g}(t-\lambda )|^2\le B,\quad \text { for a.e. }t\in {\mathbb {R}}. \end{aligned}$$

#### Proof

First, by (8) we may rotate the problem and assume $\vec {d}=\vec {e}_2$ and $\vec {d}_\bot =\vec {e}_1$. Writing the short-time Fourier transform as $V_gf(x,\xi )={\mathcal {F}}\big (f \overline{T_xg}\big )(\xi )$ and using Parseval’s identity yields

$$\begin{aligned} \int _{L_{\vec {e_2},\varLambda }}|V_gf(z)|^2\,\textrm{d}{\mathcal {H}}^1(z)&=\sum _{\lambda \in \varLambda }\int _{\mathbb {R}}|V_gf( \lambda ,\xi )|^2\,\textrm{d}\xi =\sum _{\lambda \in \varLambda }\int _{\mathbb {R}}|f(t)\overline{g(t-\lambda )}|^2\,\textrm{d}t \\&=\int _{\mathbb {R}}|f(t)|^2\left( \sum _{\lambda \in \varLambda }|g(t-\lambda )|^2\right) \,\textrm{d}t, \end{aligned}$$

where changing the order of integration and summation is allowed by either (11) or the existence of the upper sampling bound. $\square$

The uniqueness property of parallel lines on the distribution space $M^\infty _{1/\vartheta _s}({\mathbb {R}})$ can be characterized in a similar fashion.

#### Proposition 3

Let $g\in M^1_{\vartheta _s}({\mathbb {R}})\cap A_{\vartheta _s}({\mathbb {R}})$, $s\ge 0$, $\varLambda \subset {\mathbb {R}}$ be countable, and $\vec {d}=R( \theta )\vec {e}_2$. The collection of parallel lines $L_{\vec {d},\varLambda }$ is a uniqueness set for $M_{1/\vartheta _s}^\infty ({\mathbb {R}})$ if and only if

$$\begin{aligned} \bigcup _{\lambda \in \varLambda }\widetilde{supp }(T_{\lambda }U(\theta )g)={\mathbb {R}}, \end{aligned}$$

where $\widetilde{supp }(g):=\{t\in {\mathbb {R}}:\ g(t)\ne 0\}$ denotes the *effective support* of *g*.

#### Remark 2

In [6, Lemma 27], a similar result was shown for planar uniqueness sets. Since some technical details are left out there, we decided to include the proof here.

#### Proof

For $f\in M^\infty _{1/\vartheta _s}({\mathbb {R}})$, $g\in M^1_{\vartheta _s}({\mathbb {R}})\cap A_{\vartheta _s}({\mathbb {R}})$ and $h\in M^1_{\vartheta _s}({\mathbb {R}})$, we define the product *fg* via $\langle f g,h\rangle _{M^\infty _{1/\vartheta _s}\times M^1_{\vartheta _s}}$ $=\langle f,gh\rangle _{M^\infty _{1/\vartheta _s}\times M^1_{\vartheta _s}}$. By (2), it then follows that $f g\in M^\infty _{1/\vartheta _s}({\mathbb {R}})$ as

$$\begin{aligned} \big |\langle f g,h\rangle _{M^\infty _{1/\vartheta _s}\times M^1_{\vartheta _s}}\big |= & {} \big |\langle f,gh\rangle _{M^\infty _{1/\vartheta _s}\times M^1_{\vartheta _s}}\big | \\ {}\le & {} \Vert f\Vert _{M^\infty _{1/\vartheta _s}}\Vert gh\Vert _{M^1_{\vartheta _s}}\le \Vert f\Vert _{M^\infty _{1/\vartheta _s}}\Vert g\Vert _{A_{\vartheta _s}}\Vert h\Vert _{M^1_{\vartheta _s}}. \end{aligned}$$

Moreover, since $A_{\vartheta _s}({\mathbb {R}})$ is invariant under translations it follows that $f\overline{T_xg}\in M^\infty _{1/\vartheta _s}\subset {\mathcal {S}}'({\mathbb {R}})$ and consequently, ${\mathcal {F}}\big (f\overline{T_xg}\big )\in {\mathcal {S}}'({\mathbb {R}})$ is a well defined tempered distribution.

Since $M^1_{\vartheta _s}({\mathbb {R}})$ is weak-$*$ dense in $M^\infty _{1/\vartheta _s}({\mathbb {R}})$, and since the short-time Fourier transform of a distribution in $M^\infty _{1/\vartheta _s}({\mathbb {R}})$ is continuous in ${\mathbb {R}}^2$, it follows that $V_g f (x,\xi )={\mathcal {F}}\big (f\overline{ T_xg}\big )(\xi )$.

As before we may rotate the problem and assume $\vec {d}=\vec {e_2}$. Now, $V_gf|_{L_{\vec {e_2},\varLambda }}=0$ if and only if all the distributions $f\overline{T_{\lambda }g},\ \lambda \in \varLambda$, are zero. This in turn is equivalent to the support of *f* and the effective support of $T_{\lambda }g$ being disjoint for every $\lambda \in \varLambda$. $\square$

### Connection to Discrete Sampling

Proposition 2 shows that $\varGamma$ being relatively dense is not sufficient for $\varGamma$ to be a Gabor sampling trajectory for $L^2({\mathbb {R}})=M^2({\mathbb {R}})$. A natural question is therefore whether for every $g\in L^2({\mathbb {R}})\backslash \{0\}$ there exists $R^*=R^*(g)>0$ such that every $(\gamma ,R)$-dense trajectory set $\varGamma$ is a Gabor sampling trajectory if $R\le R^*$. We follow the approach of [20] to show that such $R^*$ does in fact exist for a certain class of window functions.

Let us write $Q_R(z):=Rz+ [-R/2,R/2)^2$, $Xg(t)=t g(t)$, and recall the definition of the Sobolev space $H^1({\mathbb {R}}):=\Big \{f\in L^2({\mathbb {R}}):\ \int _{\mathbb {R}}(1+|\xi |^2)|{\widehat{f}}(\xi )|^2\,\textrm{d}\xi <\infty \Big \}.$

#### Proposition 4

Let $g,tg\in H^1({\mathbb {R}})$, $R>0$ be chosen such that

12 $$\begin{aligned} \varDelta :=\frac{2R}{\pi }\left( \Vert g'\Vert _2+\Vert Xg\Vert _2+\frac{2R}{\pi }\Vert Xg'\Vert _2\right) <\Vert g\Vert _2, \end{aligned}$$

and $\varGamma \subset {\mathbb {R}}^2$ be a trajectory set. If there exist $m,M>0$ such that

13 $$\begin{aligned} 0<m\le {\mathcal {H}}^1(\varGamma \cap Q_R(z))\le M<\infty ,\quad \text {for every }z\in {\mathbb {R}}^2, \end{aligned}$$

then for every $f\in L^2({\mathbb {R}})$

$$\begin{aligned} m\big (\Vert g\Vert _2-\varDelta \big )^2\Vert f\Vert ^2\le \int _\varGamma |V_gf (z)|^2 \,\textrm{d}{\mathcal {H}}^1(z)\le M\big (\Vert g\Vert _2+\varDelta \big )^2\Vert f\Vert ^2. \end{aligned}$$

#### Proof

It is shown in [35] that, for the particular choice of *g* and *R*, arbitrary points $z_{n}\in Q_R(n),\ n \in {\mathbb {Z}}^2$, generate a discrete frame $\{\pi (z_{n})g\}_{n\in {\mathbb {Z}}^2}$ for $L^2({\mathbb {R}})$ with uniform frame bounds $A=\big (\Vert g\Vert _2-\varDelta \big )^2$ and $B=\big (\Vert g\Vert _2+\varDelta \big )^2$.

For every $n \in {\mathbb {Z}}^2$ there exists $z_{n }\in \varGamma \cap Q_R({n})$ such that

$$\begin{aligned} |V_g f(z_{n})|^2\ge \frac{1}{{\mathcal {H}}^1(\varGamma \cap Q_R({n}))}\int _{\varGamma \cap Q_R({n})}|V_g f(z)|^2\,\textrm{d}{\mathcal {H}}^1(z). \end{aligned}$$

Then, as every choice of points $z_{n }\in Q_R({n})$ generates a Gabor frame with uniform upper bound *B*, we have

$$\begin{aligned} \int _\varGamma |V_{g}f(z)|^2\,\textrm{d}{\mathcal {H}}^1(z)&=\sum _{n\in {\mathbb {Z}}^2}\int _{\varGamma \cap Q_R({n})} |V_{g}f(z)|^2\,\textrm{d}{\mathcal {H}}^1(z) \\&\le \sum _{n\in {\mathbb {Z}}^2}{\mathcal {H}}^1(\varGamma \cap Q_R({n})) |V_{g}f(z_{n})|^2\le MB \Vert f\Vert ^2. \end{aligned}$$

The lower sampling bound follows with a similar argument. $\square$

#### Remark 3

This construction works for general measures and gives a characterization of sampling measures for this class of window functions.

As $Q_R(z)\subset B_{\sqrt{2}R}(z)\subset Q_{2R}(z)$, it follows that (13) implies $m\le {\mathcal {H}}^1(\varGamma \cap B_{\sqrt{2}R}(z))\le 4M$, for every $z\in {\mathbb {R}}^2,$ that is, $\varGamma$ is relatively dense in the sense of Sect. 3.1. The quantitative estimate of the frame bounds however depends on the relation (13).

## Spiraling Curves

### Weak Limits

There are multiple ways of defining weak limits of trajectory sets. The definition in [21], for example, adapts the original notion by Beurling [11] given in terms of a geometric condition. For our purposes, it will be more convenient to work with a stronger notion that was introduced to define weak limits of measures, see [6].

#### Definition 1

Let $\varGamma ,\varGamma ',\varGamma _n$ be trajectory sets in ${\mathbb {R}}^2$. We say that $\{\varGamma _n\}_{n\in {\mathbb {N}}}$ *converges weakly* to $\varGamma$ if

$$\begin{aligned} \int _{\varGamma _n}\phi (z) \,\textrm{d}{\mathcal {H}}^1(z)\rightarrow \int _{\varGamma }\phi (z) \,\textrm{d}{\mathcal {H}}^1(z), \end{aligned}$$

for every nonnegative function $\phi \in C_c({\mathbb {R}}^2)$. In that case we write $\varGamma _n{\mathop {\rightarrow }\limits ^{w}}\varGamma$.

We say that $\varGamma '$ is a weak limit of translates of $\varGamma$ if there exists a sequence $\{z_n\}_{n\in {\mathbb {N}}}$ such that $z_n+\varGamma {\mathop {\rightarrow }\limits ^{w}}\varGamma '$ and define ${\mathcal {W}}_\varGamma$ as the set of all weak limits of translates of $\varGamma$.

The following characterization of Gabor sampling trajectories is an immediate consequence of a characterization of samping measures for the short-time Fourier transform given by Ascensi [6, Theorem 14]. One can think of this result as a time-frequency analog of the classical result by Beurling [11, Theorem 3, p. 345] where the Gabor sampling property on $M^p({\mathbb {R}})$ is connected to the uniqueness property of all weak limits of translates on the larger space $M^p_{1/\vartheta _s}({\mathbb {R}})$. Note that Ascensi’s result requires a special class of windows which we define in a simplified version taylored to polynomial weights.

#### Definition 2

We say that $1/\vartheta _s$ *controls* $V_gg$ if there exists a decreasing function $d:{\mathbb {R}}^+\rightarrow {\mathbb {R}}^+$ with $d(r)\rightarrow 0$, as $r\rightarrow \infty$, such that $|V_gg(z)|\le d(|z|)/\vartheta _s(z).$ The class ${\mathcal {M}}({\mathbb {R}})$ is then given by

$$\begin{aligned} {\mathcal {M}}({\mathbb {R}}):=\big \{g\in L^2({\mathbb {R}}):\ \exists s>2\text { s.t. } 1/\vartheta _s \text { controls }V_gg\big \}. \end{aligned}$$

Note that ${\mathcal {M}}({\mathbb {R}})$ contains, for example, functions in the Schwartz class as well as window functions considered in the theory of intrinsically localized frames [14].

#### Theorem 3

(Ascensi) Let $g\in {\mathcal {M}}({\mathbb {R}})$, $s>2$ be such that $1/\vartheta _s$ controls $V_gg$, and $1\le p<\infty$. Then $\varGamma$ is a Gabor sampling trajectory for $M^p({\mathbb {R}})$ if and only if every $\varGamma '\in {\mathcal {W}}_\varGamma$ is a set of uniqueness for $M^p_{1/\vartheta _s}({\mathbb {R}})$.

### Spiraling Curves and Their Weak Limits

The notion of a spiraling curve was introduced in [21]. This class of trajectory sets includes a wide range of natural examples such as the concentric circles or the Archimedes spiral. In this paper, we use a slightly more restrictive notion of spiraling curves that still includes the main examples from [21] while allowing for a full characterization of the set of weak limits of translates.

#### Definition 3

(*Spiraling curve*) Let ${\mathcal {I}}_\varGamma \subset {\mathbb {T}}$ be a finite set. A trajectory set $\varGamma$ is called *spiraling* if the following conditions are satisfied:

If $$\beta \in {\mathbb {T}}\backslash {\mathcal {I}}_\varGamma $$, then

(A.i) (*Escape Cone*) for $\alpha :=\min \{\text {dist}(\beta ,{\mathcal {I}}_\varGamma ),1/8\}$ the intersection of $\varGamma$ with the cone
  $$\begin{aligned} {\mathcal {C}}_{\alpha ,\beta }:=\{(r \cos ( 2\pi \theta ),r \sin (2\pi \theta )):\ r\ge 0,\ \beta -\alpha \le \theta \le \beta +\alpha \} \end{aligned}$$
  can be parametrized in polar coordinates as
  $$\begin{aligned} \gamma _\beta (\theta )=(r_\beta (\theta )\cos ( 2\pi \theta ),r_\beta (\theta )\sin (2\pi \theta )) \end{aligned}$$
  with $\theta \in \bigcup _{k\in {\mathbb {N}}} [k+\beta -\alpha ,k+\beta +\alpha ]$ and $r_\beta$ a nonnegative $C^2$-function on each interval whose $C^2$-norm is globally bounded.
(A.ii) (*Asymptotic radial monotonicity*) there exists $K\in {\mathbb {N}}$ such that for any $\theta \in [ -\alpha , \alpha ]$ fixed, the sequence $r_\beta (k+\beta +\theta )$ is strictly increasing for $k\ge K$.
(A.iii) (*Asymptotic flatness*) the curvature of $\gamma _\beta$, denoted by $\kappa _\beta$, tends to 0 as its input goes to infinity. To be more precise, we assume
  14 $$\begin{aligned} \sup _{\theta \in (-\alpha ,\alpha )} \kappa _\beta (k+\beta +\theta )\rightarrow 0,\quad k\rightarrow \infty . \end{aligned}$$
(A.iv) (*Asymptotic equispacing*) there exist continuous functions $\eta _\beta :[-\alpha ,\alpha ]\rightarrow {\mathbb {R}}^+,\rho _\beta :[-\alpha ,\alpha ]\rightarrow {\mathbb {R}}$ such that
  $$\begin{aligned} \sup _{\theta \in [-\alpha ,\alpha ]}\left| r_{ \beta }(k+\beta +\theta )-\eta _\beta (\theta )k -\rho _\beta (\theta )\right| \rightarrow 0,\quad k\rightarrow \infty . \end{aligned}$$
(A.v) (*Asymptotic velocity*) there exists a continuous function $\vec {d}_\beta :[ -\alpha , \alpha ]\rightarrow {\mathbb {S}}^1$ such that $\vec {d}_\beta (\theta )$ is non-collinear with $\vec {\ell }(\theta )$ and
  15 $$\begin{aligned} \sup _{\theta \in [-\alpha ,\alpha ]}\left\| \frac{\gamma _\beta ^\prime (k+\beta +\theta )}{\Vert \gamma _\beta ^\prime (k+\beta +\theta )\Vert } -\vec {d}_\beta (\theta )\right\| \rightarrow 0,\quad k\rightarrow \infty . \end{aligned}$$

If $\beta \in {\mathcal {I}}_\varGamma$, then $(A.i)-(A.v)$ hold with the following modifications

(B.i) for $\alpha :=\min \{dist (\beta ,{\mathcal {I}}_\varGamma \backslash \{\beta \}),1/8\}$, the parametrization $\gamma _\beta |_{[k+\beta -\alpha ,k+\beta +\alpha ]}$ is continuous and the restrictions of $\gamma _\beta$ to ${(k+\beta ,k+\beta +\alpha ]}$ and ${[k+\beta -\alpha ,k+\beta )}$ are $C^2$-functions with bounded $C^2$-norms.
(B.iii) The supremum in (14) is replaced by an supremum over $(-\alpha ,\alpha )\backslash \{0\}$.
(B.v) The velocity vector $\vec {d}_\beta$ is continuous on $[ -\alpha ,\alpha ]\backslash \{0\}$. Morover, $\lim _{\theta \nearrow 0}\vec {d}_\beta (\theta )$ and $\lim _{\theta {\searrow }0}\vec {d}_\beta (\theta )$ exist, and the supremum in (15) is taken over $[-\alpha ,\alpha ]\backslash \{0\}$.

#### Remark 4

(i) The original definition of spiraling curves [21, Sect. 3.3] only assumed that there exists at least one angle such that the conditions $(A.i)-(A.v)$ are satisfied. Let us mention here that our additional assumptions are also met by the examples mentioned in [21]. In particular, the set of concentric circles is also a spiraling curve in the sense of Definition 3.
(ii) Since the collection of star shaped polygons and paths (as defined in Theorem 1) generated by a set of points consist of countably many line segments (mostly parallel and equispaces within escape cones), it is a straightforward task to show that these trajectory sets are indeed spiraling curves. The only escape cones that need more attention are those intersecting the line segments $s({kz_N, (k+1)z_1})$. In the limit however, these are parallel and equispaced line segments.

Subsequently, we give a full characterization of the set of all weak limits of translates of spiraling curves. To do so, we establish two technical lemmas that describe the weak limits of $z_k+\varGamma$ according to a certain geometric condition on the sequence $\{z_k\}_{k\in {\mathbb {N}}}$.

#### Lemma 2

Let $z_k = - r_k \vec {\ell }(\theta _k),\ k\in {\mathbb {N}},$ be an unbounded sequence. If there exist no pair $({\mathcal {N}},\gamma )$, ${\mathcal {N}}\subset {\mathbb {N}},\ \gamma \in {\mathcal {I}}_\varGamma$, such that

$$\begin{aligned} \mathrm{(i)}\,\, \{r_k\}_{k\in {\mathcal {N}}}\text { is unbounded\;and}\;\mathrm{(ii)}\,\, \{r_{k}\sin (2\pi (\theta _{k}-\gamma ))\}_{k \in {\mathcal {N}}}\;\text {is bounded,} \end{aligned}$$

then there exist a subsequence $\{z_{k_n}\}_{n\in {\mathbb {N}}}$, an angle $\beta \in {\mathbb {T}}\backslash {\mathcal {I}}_\varGamma$, and a constant $\tau \in {\mathbb {R}}$, such that (a) $-z_{k_n}\in Int ({\mathcal {C}}_{\alpha ,\beta })$, for every $n\in {\mathbb {N}}$, (b) $\theta _{k_n}\rightarrow \theta ^*$, $n\rightarrow \infty$, and

$$\begin{aligned} \mathrm{(c)}\;z_{k_n}+\varGamma {\mathop {\rightarrow }\limits ^{w}}\tau \vec {d}_\bot +L_{\vec {d},\lambda {\mathbb {Z}}}, \end{aligned}$$

where $$\vec {d}=\lim _{n\rightarrow \infty } \vec {d}_\beta (\beta -\theta _{k_n})$$ and $$\lambda = \eta _\beta (\beta -\theta ^*) \sin \big (\arccos \big (\vec {\ell }(\theta ^*) \cdot \vec {d}\ \big )\big ).$$

Moreover, for every $\tau \in {\mathbb {R}}$, $\beta \in {\mathbb {T}}\backslash {\mathcal {I}}_\varGamma$ and for $\vec {d}=\lim _{\theta \searrow 0}\vec {d}_{\beta }(\theta )$ as well as for $\vec {d}=\lim _{\theta \nearrow 0}\vec {d}_{\beta }(\theta )$, there exist a sequence $\{z_k\}_{k\in {\mathbb {N}}}$ such that

$$\begin{aligned} z_k+\varGamma {\mathop {\rightarrow }\limits ^{w}}\tau \vec {d}_\bot +L_{\vec {d},\lambda {\mathbb {Z}}}, \end{aligned}$$

with $$\lambda = \eta _\beta (0) \sin \big (\arccos \big (\vec {\ell }(\beta )\cdot \vec {d}\big )\big )$$.

#### Remark 5

The distinction of the two directions $\vec {d}=\lim _{\theta \searrow 0}\vec {d}_{\beta }(\theta )$ and $\vec {d}=\lim _{\theta \nearrow 0}\vec {d}_{\beta }(\theta )$ (which coincide for $\beta \in {\mathbb {T}}\backslash {\mathcal {I}}_\varGamma$) in Lemma 2 is necessary as, for $\beta \in {\mathcal {I}}_\varGamma$, the sets of parallel lines with the left and right limits of the asymptotic velocity as directions are both included in ${\mathcal {W}}_\varGamma$.

#### Proof

*Step 1*   Since $r_k\sin (2\pi (\theta _k-\gamma ))$ is unbounded for every $\gamma \in {\mathcal {I}}_\varGamma$ and since ${\mathbb {T}}$ is compact we can, after passing to a subsequence, assume that $\{-z_k\}_{k\in {\mathbb {N}}}$ is contained in the interior of an escape cone ${\mathcal {C}}_{\alpha ,\beta }$ for some $\beta \in {\mathbb {T}}\backslash {\mathcal {I}}_\varGamma$ and that $\theta _k\rightarrow \theta ^*\in [-\alpha +\beta ,\alpha +\beta ]$.

As the problem is rotation invariant, we may for simplicity assume that $\beta =0$. In the following we therefore omit the subscripts in $\eta _\beta , r_\beta$, etc.. As $\Vert z_k\Vert$ is unbounded, we can, after passing to yet another subsequence, write $z_k$ as

$$\begin{aligned} z_k= -\big (\eta (\theta ^*)(n_k+v_k)+\rho (\theta ^*)\big )\vec {\ell }( \theta _k), \end{aligned}$$

where $n_k\in {\mathbb {N}}$ is strictly increasing, $v_k\in [0,1]$ converges to $v^*$, and $\eta$, $\rho$ are the functions given by (A.iv). If $z_k+\varGamma$ converges weakly to $\varGamma '$, then $z_k+v_k\eta (\theta ^*)\vec {\ell }(\theta _k)+\varGamma$ converges weakly to $v^*\eta (\theta ^*)\vec {\ell }(\theta ^*)+\varGamma '$. As $z_k+v_k\eta (\theta ^*)\vec {\ell }(\theta _k)$ still satisfies the assumption of this lemma, we may further simplify notation and assume

$$\begin{aligned} z_k=-(\eta (\theta ^*)k+\rho (\theta ^*))\vec {\ell }(\theta _k). \end{aligned}$$

*Step 2*  We will later (in Step 4) show that the following property is always satisfied: For every compact set $K\subset {\mathbb {R}}^2$ there exists $k^*\in {\mathbb {N}}$ such that

$$\begin{aligned} K\subset z_k+{\mathcal {C}}_{\alpha -|\theta _k|,\theta _k},\quad k\ge k^*. \end{aligned}$$

For $h\in C_c({\mathbb {R}}^2)$, there exist $T_\theta ,S_\theta >0$ and $t_\theta \in {\mathbb {R}}$ such that the parallelogram

$$\begin{aligned} {\widetilde{P}}_{\theta }=\big \{(t_\theta +t)\vec {\ell }({\theta })+s\vec {d}(\theta ): \ s\in [-S_\theta ,S_\theta ],\ t\in [0,T_\theta ]\big \} \end{aligned}$$

satisfies $\text {supp}(h)\subset {\widetilde{P}}_{\theta }$. Note that $T_\theta ,S_\theta$ can be chosen to depend continuously on $\theta \in [-\alpha ,\alpha ]$. In order to simplify the argument, we define a slightly larger parallelogram $P_{\theta }$ containing ${\widetilde{P}}_{\theta }$. To this end, let $m_{\theta },M_{\theta }\in {\mathbb {Z}}$ be the largest (respectively smallest) integer such that $m_{\theta }\eta (\theta )\le t_\theta$, and $M_{\theta } \eta (\theta ) \ge t_\theta +T_\theta$ and define

$$\begin{aligned} P_{\theta }=\Big \{ (m_{\theta }-1/2+t)\eta (\theta ) \vec {\ell }(\theta )+s\vec {d}(\theta ): \ s\in [-S_\theta ,S_\theta ],\ t\in [0,M_{\theta }-m_{\theta }+1]\Big \}, \end{aligned}$$

as well as the smaller parallelograms

$$\begin{aligned} P_{\theta ,m}=\Big \{ (m-1/2+t)\eta (\theta ) \vec {\ell }(\theta )+s\vec {d}(\theta ): \ s\in [-S_\theta ,S_\theta ],\ t\in [0,1]\Big \},\quad m_{\theta }\le m\le M_{\theta }. \end{aligned}$$

Since all parameters defining $P_\theta$ are continuously depending on $\theta$, it first follows that the number smaller parallelograms is bounded by $\max _{\theta \in [-\alpha ,\alpha ]} T_\theta /\eta (\theta )+2$. Secondly, there exists a compact set *K* such that $P_\theta \subset K$ for ever $\theta \in [-\alpha ,\alpha ]$. Consequently, by our previous assumption, for *k* large enough we have that ${P}_{\theta _k }\subset K \subset z_k+C_{\alpha -|\theta _k|,\theta _k }$.

*Step 3*  For each *k*, *m*, let $\psi _{k,m}:I_{k,m}\rightarrow {\mathbb {R}}^2$ be a re-parametrization by arc-length of the segment $\{z_k+\gamma (k+m+\theta )\}_{\theta \in [-\alpha ,\alpha ]}$ such that

$$\begin{aligned} \psi _{k,m}(0)=z_k+\gamma (k+m+\theta _k)= \big (r(k+m+\theta _k) -k\eta (\theta ^*)-\rho (\theta ^*)\big )\vec {\ell }(\theta _k). \end{aligned}$$

For large values of *k*, $\psi _{k,m}(0)$ approximates $m\eta (\theta ^*)\vec {\ell }(\theta ^*)$. Applying a first order Taylor approximation yields

16 $$\begin{aligned}{} & {} \big \Vert \psi _{k,m}(t)-\big (r(k+m+\theta _k) -k\eta (\theta ^*)-\rho (\theta ^*)\big )\vec {\ell }(\theta _k)-t\psi '_{k,m}(0)\big \Vert \nonumber \\ {}{} & {} \quad \le \frac{t^2}{2}\sup _{s\in I_{k,m}}\Vert \psi _{k,m}''(s)\Vert . \end{aligned}$$

Let us define $S_{\max }:= \max _{\theta \in [-\alpha ,\alpha ]}S_\theta$ (and $S_{\min }$ analogously). By conditions (A.iii)-(A.v) of Definition 3 and

$$\begin{aligned} \sup _{s\in I_{k,m}}\Vert \psi _{k,m}''(s)\Vert = \sup _{\theta \in [-\alpha ,\alpha ]} |\kappa (k+m+\theta )|\rightarrow 0, \quad \text {as }k\rightarrow \infty , \end{aligned}$$

we have that for every $\delta >0$ there exists $k^*\in {\mathbb {N}}$ such that

(i) $$|\kappa (k+m+\theta )|<\delta /8S_{\max }^2$$,
(ii) $$ \big \Vert \psi ^\prime _{k,m}(0)-\vec {d}(\theta _k)\big \Vert =\left\| \frac{\gamma ^\prime (k+m+\theta _k)}{\Vert \gamma ^\prime (k+m+\theta _k) \Vert }-\vec {d}(\theta _k)\right\| < {\delta }/{8S_{\max }}, $$
(iii) $$|r({k+m}+\theta ^*)-\eta (\theta ^*)(k+m)-\rho (\theta ^*)|<\delta /4$$,
(iv) $$|r({k+m}+\theta _k)-r(k+m+\theta ^*)|<\delta /4$$,

whenever $k\ge k^*$. Note that we have used that the $C^2$-norm of *r* is globally bounded to ensure the last estimate. Now, if $t\in [-2 S_{\max },2S_{\max }]$ and $k\ge k^*$, then, using (16) and triangle inequality, we get

$$\begin{aligned}&\big \Vert \psi _{k,m}(t)- \eta (\theta ^*)m\vec {\ell }(\theta _k)-t\vec {d}(\theta _k)\big \Vert \\&\le |r(k+m+\theta ^*)-r(k+m+\theta _k)|\\&\quad +|r(k+m+\theta ^*)-\eta (\theta ^*)(k+m)-\rho (\theta ^*)| \\&\quad + |t|\Vert \psi '_{k,m}(0)-\vec {d}(\theta _k)\Vert + \frac{t^2}{2}\sup _{s\in I_{k,m}}\Vert \psi _{k,m}''(s)\Vert \\&< \delta /4+\delta /4+\delta /4+\delta /4=\delta . \end{aligned}$$

Hence, if $\delta <\min \{S_{\min },\eta \}$, then $\psi _{k,m}(I_{k,m})\cap P_{\theta _k}\subset P_{\theta _k,m}$, and $\psi _{k,m}(t) \notin P_{\theta _k,m}$ for every $|t|\ge 2S_{\max }$.

As *h* is uniformly continuous we may choose $\delta$ according to $\varepsilon$ such that $\Vert x-y\Vert <\delta$ implies $|h(x)-h(y)|<\varepsilon$. Let $\lambda _k=\eta (\theta _k)\sin \big (\arccos (\vec {\ell }(\theta _k)\cdot \vec {d}(\theta _k))\big )$. Then, for $k\ge k^*$

$$\begin{aligned}&\left| \int _{z_k+\varGamma }h(x)\,\textrm{d}{\mathcal {H}}^1(x)-\int _{L_{\vec {d}(\theta _k),\lambda _k{\mathbb {Z}}}}h(x)\,\textrm{d}{\mathcal {H}}^1(x)\right| \\&\quad = \sum _{m=m_{ \theta _k}}^{M_{ \theta _k}}\left| \int _{(z_k+\varGamma )\cap P_{\theta _k,m}}h(x)\,\textrm{d}{\mathcal {H}}^1(x)-\int _{L_{\vec {d}(\theta _k),\lambda _k{\mathbb {Z}}}\cap P_{\theta _k,m}}h(x)\,\textrm{d}{\mathcal {H}}^1(x)\right| \\&\quad \le \sum _{m=m_{\theta _k}}^{M_{ \theta _k}}\int _{-2S_{\max }}^{2S_{\max }}\left| h(\psi _{k,m}(t)) -h\big (\eta (\theta _k)m\vec {\ell }({\theta _k})+\vec {d}(\theta _k)t\big )\right| \,\textrm{d}t \\&\quad \le 4 S_{\max } \sum _{m=m_{ \theta _k}}^{M_{ \theta _k}} \sup _{t\in [-2S_{\max },2S_{\max }]}\left| h(\psi _{k,m}(t)) -h\big ( \eta (\theta _k)m\vec {\ell }({\theta _k})+\vec {d}(\theta _k)t\big )\right| \\&\quad \le 4S_{\max } (M_{ \theta _k}-m_{ \theta _k})\varepsilon \le 4S_{\max } \left( \max _{\theta \in [-\alpha ,\alpha ]} T_\theta /\eta (\theta )+2\right) \varepsilon . \end{aligned}$$

As $\theta _k\rightarrow \theta ^*$ and consequently $\lambda _k\rightarrow \lambda ^*$, it is clearly possible to find $k^*$ such that

$$\begin{aligned} \left| \int _{ L_{\vec {d}(\theta ^*),\lambda ^*{\mathbb {Z}}}}h(x)\,\textrm{d}{\mathcal {H}}^1(x)-\int _{ L_{\vec {d}(\theta _k),\lambda _k{\mathbb {Z}}}}h(x)\,\textrm{d}{\mathcal {H}}^1(x)\right| \le \varepsilon , \end{aligned}$$

whenever $k\ge k^*$. Therefore, by triangle inequality, the convergence $z_k+\varGamma {\mathop {\rightarrow }\limits ^{w}} L_{\vec {d}(\theta ^*),\lambda ^*{\mathbb {Z}}}$ follows.

*Step 4* It remains to show that the assumption $K\subset z_k+{\mathcal {C}}_{\alpha -|\theta _k|,\theta _k}$ can always be satisfied for *k* large enough. To this end, we distinguish the cases $|\theta ^*|<\alpha$ and $|\theta ^*|=\alpha$. In the former case, we set $\alpha ^*=(\alpha -|\theta ^*|)/2$ and observe that for *k* large enough $-\frac{1}{2}\Vert z_k\Vert \vec {\ell }(\theta ^*)+ {\mathcal {C}}_{\alpha ^*,\theta ^*}\subset z_k+ {\mathcal {C}}_{\alpha -|\theta _k|,\theta _k}$ and the left side of the inclusion relation will eventually cover any compactum.

If $|\theta ^*|=\alpha$ and $\theta ^*\notin {\mathcal {I}}_\varGamma$, then it is possible to apply a rotation by a small angle such that $\alpha$ is still the opening angle for the escape cone of $\beta =0$ and $|\theta ^*|<\alpha$.

**Fig. 2:**
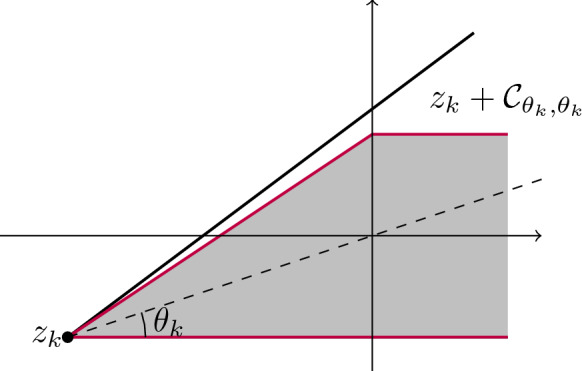
The set from the left hand side of (17) (gray shaded area) and the cone $z_k+{\mathcal {C}}_{\theta _k,\theta _k}$

If $|\theta ^*|=\alpha$ and $\theta ^*\in {\mathcal {I}}_\varGamma$, then one can rotate the problem so that $\theta ^*=0$. Moreover, we can without loss of generality assume that $\theta _k>0$. Let us consider the cone ${\mathcal {C}}_{\theta _k,\theta _k}$. A simple geometric argument shows that

17 $$\begin{aligned} \big \{(x,y)\in {\mathbb {R}}^2:\ x\ge -a_k,\ |y|\le b_k,\text { and } {2}b_k x+a_kb_k\ge a_ky \big \}\subset z_k+{\mathcal {C}}_{\theta _k,\theta _k},\nonumber \\ \end{aligned}$$

where $a_k=r_k\cos (2\pi \theta _k)$ and $b_k=r_k\sin (2\pi \theta _k)$, see Fig. 2 for an illustration.

The assumptions that there is no unbounded subsequence of $\{z_k\}_k$ such that $r_{k_n}\sin (2\pi \theta _{k_n})$ is bounded and $|\theta _k|\le \alpha \le 1/8$ then shows, after possibly passing to yet another subsequence, that $z_k+{\mathcal {C}}_{\theta _k,\theta _k}$ eventually covers any compactum.

*Step 5* To see that every such collection of parallel lines is in fact a weak limit, we may assume that $\vec {d}=\lim _{\theta \searrow 0}\vec {d}_{\beta }(\theta )$ ($\vec {d}=\lim _{\theta \nearrow 0}\vec {d}_{\beta }(\theta )$ works exactly the same) and choose

$$\begin{aligned} z_k=\tau \vec {d}_\bot -(\eta _\beta (0)k+\rho _\beta (0))\vec {\ell }\big (\beta +1/\sqrt{k}\big ),\ \beta \in {\mathbb {T}}\backslash {\mathcal {I}}_\varGamma . \end{aligned}$$

For this choice, the assumptions of the first part of this lemma are satisfied and one may repeat the arguments to show that $z_k+\varGamma {\mathop {\rightarrow }\limits ^{w}}\tau \vec {d}_\bot +L_{\vec {d},\eta {\mathbb {Z}}}$. $\square$

#### Lemma 3

Let $z_k = -r_k \vec {\ell }(\theta _k)$, $k\in {\mathbb {N}}$, be an unbounded sequence. If there exists a pair $({\mathcal {N}},\gamma ),\ {\mathcal {N}}\subset {\mathbb {N}}$, $\gamma \in {\mathcal {I}}_\varGamma$, such that

$$\begin{aligned} \mathrm{(i)}\,\, \{r_k\}_{k \in {\mathcal {N}}}\text { is unbounded and } \mathrm{(ii)}\,\, \{r_{k}\sin (2\pi (\theta _{k}-\gamma ))\}_{k \in {\mathcal {N}}}\text { is bounded,} \end{aligned}$$

then there exist a subsequence $\{z_{k_n}\}_{n\in {\mathbb {N}}}$, and $z\in {\mathbb {R}}^2$, such that

$$\begin{aligned} z_{k_n}+\varGamma {\mathop {\rightarrow }\limits ^{w}} z+E_{\gamma }, \end{aligned}$$

where the set of *parallel edges* $E_\gamma$ is given by

$$\begin{aligned} E_{\gamma }:= & {} \left\{ \eta _\gamma k\vec {\ell }(\gamma )-t\vec {d}_{\gamma }^{-}:\ k\in {\mathbb {Z}},\ t\in [0,\infty )\right\} \\ {}{} & {} \quad \cup \left\{ \eta _\gamma k\vec {\ell }(\gamma )+t\vec {d}_\gamma ^+:\ k\in {\mathbb {Z}},\ t\in [0,\infty )\right\} , \end{aligned}$$

and $$\eta _\gamma =\eta _\gamma (0)$$, $$\vec {d}_\gamma ^-=\lim _{\theta \nearrow 0} \vec {d}_\gamma (\theta )$$, and $$\vec {d}_\gamma ^+=\lim _{\theta \searrow 0} \vec {d}_\gamma (\theta )$$.

Moreover, for every $\gamma \in {\mathcal {I}}_\varGamma$ and every $z\in {\mathbb {R}}^2$ there exist a sequence $\{z_k\}_{k\in {\mathbb {N}}}$ such that $z_k+\varGamma {\mathop {\rightarrow }\limits ^{w}}z+E_\gamma$.

#### Proof

After potentially passing to a subsequence, one can assume that ${\mathcal {N}}={\mathbb {N}}$, and that $r_k$ is an increasing unbounded sequence. Moreover, by rotation invariance we may set $\gamma =0$. By assumption, the sequence $r_k\sin (2\pi \theta _k)$ is bounded which shows that there exists a subsequence converging to $y^*$. Therefore, after passing to this subsequence, we see that if $z_k+\varGamma$ converges to $\varGamma '$, then $(-r_k\cos \theta _k,0)+\varGamma$ converges to $(0,-y^*)+\varGamma '$. Therefore, we further simplify the problem and assume that $z_k=(-{\widetilde{r}}_k,0)$. From here we can basically proceed as in the proof of Lemma 2 with some minor adjustments.

Let us shortly point out where caution is needed. For $h\in C_c({\mathbb {R}}^2)$ the parallelograms ${\widetilde{P}}_0$ need to be replaced by arrow shaped objects defined as follows: let $t^*\in {\mathbb {R}}$ and $S,T>0$ be chosen such that the set

$$\begin{aligned}{} & {} {\widetilde{A}}_{0}:=\big \{(t^*+t)\vec {e}_1-s\vec {d}_0^-:\ t\in [0,T], s\in [0,S]\big \} \ \cup \\{} & {} \quad \big \{(t^*+t)\vec {e}_1+s\vec {d}_0^+:\ t\in [0,T], s\in [0,S]\big \} \end{aligned}$$

contains $\text {supp}(h)$. Then one can proceed almost exactly as before by replacing $P_0$ by $A_0$, using the assumptions $(B.iii)-(B.v)$ of Definition 3 and applying two Taylor expansions for the left and right limits.

Again, we are left with showing that each such set of parallel edges is indeed a weak limit of translates. Setting $z_k=z-(\eta _\gamma (0)k+\rho _\gamma (0))\vec {\ell }(\gamma )$, $\gamma \in {\mathcal {I}}_\varGamma$, however yields that $z_k+\varGamma {\mathop {\rightarrow }\limits ^{w}} z+E_\gamma$. $\square$

#### Theorem 4

The set of weak limits of translates of a spiraling curve $\varGamma$ is given by

18 $$\begin{aligned} {\mathcal {W}}_\varGamma ={\mathcal {S}}_\varGamma \cup {\mathcal {L}}_\varGamma \cup {\mathcal {E}}_\varGamma , \end{aligned}$$

where $${\mathcal {S}}_\varGamma =\{z+\varGamma :\ z\in {\mathbb {R}}^2\}$$, $${\mathcal {E}}_\varGamma = \big \{z+E_{\beta }:\ \beta \in {\mathcal {I}}_\varGamma ,\ z\in {\mathbb {R}}^2\big \}$$, and

$$\begin{aligned} {\mathcal {L}}_\varGamma =\Big \{\tau \vec {d}_\bot +L_{\vec {d},\lambda {\mathbb {Z}}}:\ \tau \in {\mathbb {R}},\ \vec {d}=\lim _{\theta \searrow 0}\vec {d}_{\beta }(\theta ),\text { or }\ \vec {d}=\lim _{\theta \nearrow 0}\vec {d}_{\beta }(\theta ),\ \beta \in {\mathbb {T}}\Big \}, \end{aligned}$$

and $\lambda$ is defined as in Lemma 2.

#### Proof

Lemmas 2 and 3 imply that ${\mathcal {S}}_\varGamma \cup {\mathcal {L}}_\varGamma \cup {\mathcal {E}}_\varGamma \subseteq {\mathcal {W}}_\varGamma$.

Now let $z_k+\varGamma {\mathop {\rightarrow }\limits ^{w}}\varGamma '$. If $\Vert z_{k}\Vert \le C$, then there exist a converging subsequence $z_{k_n}\rightarrow z^*$ and it follows that $\varGamma '=z^*+\varGamma \in {\mathcal {S}}_\varGamma$. If $\{z_k\}_{k\in {\mathbb {N}}}$ is unbounded, then either there exist ${\mathcal {N}}\subset {\mathbb {N}}$ and $\gamma \in {\mathcal {I}}_\varGamma$ such that $\{z_k\}_{k\in {\mathcal {N}}}$ is unbounded and $\{r_k \sin (2\pi (\theta _k-\gamma ))\}_{k\in {\mathcal {N}}}$ is bounded, or not. Hence, the assumption of either Lemma 2 or Lemma 3 are satisfied which leaves us with the limit $\varGamma '$ being either a set of parallel lines or a set of parallel edges, i.e. ${\mathcal {W}}_\varGamma \subseteq {\mathcal {S}}_\varGamma \cup {\mathcal {L}}_\varGamma \cup {\mathcal {E}}_\varGamma$. $\square$

### Spiraling Curves as Gabor Sampling Trajectories

After fully characterizing the set of all weak limits of translates we can now prove our main results. We first show that, for a certain class of windows, spiraling curves are Gabor sampling trajectories if and only if they are uniqueness sets for the weighted modulation space $M^p_{1/\vartheta _s}({\mathbb {R}})$. Later, we then verify that certain spiraling curves are indeed such uniqueness sets.

#### Theorem 5

Let $\varGamma \in {\mathbb {R}}^2$ be a spiraling curve, $g\in \text {span}\{h_n: n\in {\mathbb {N}}_0\}$, and $1\le p<\infty$. Then $\varGamma$ is a Gabor sampling trajectory for $M^p({\mathbb {R}})$ if and only if $\varGamma$ is a uniqueness set for $M^p_{1/\vartheta _s}({\mathbb {R}})$ for some $s>2$.

To show this theorem we first need to state two auxiliary lemmas. The first one is a consequence of [16, Corollary 3.9(c) & Proposition 2.2].

#### Lemma 4

If there exist $\alpha >0$ such that $|g(t)|\lesssim e^{-\alpha |t|}$ and $|{\widehat{g}}(\xi )|\lesssim e^{- \alpha |\xi |}$, then $|V_gg(z)|\lesssim {1}/{\vartheta _{s}(z)}$, for every $s>0$.

The following lemma is a simple consequence of [6, Corollary 26].

#### Lemma 5

Let $g\in \text {span}\{h_n: n\in {\mathbb {N}}_0\}$. For every $f\in M^p_{1/\vartheta _s}({\mathbb {R}})$, $1\le p<\infty$, $z\in {\mathbb {R}}^2$, and $\beta \in {\mathbb {T}}$, it holds that $V_{g}f(z+t\vec {\ell }(\beta ))$ is a real analytic function in $t\in {\mathbb {R}}$.

#### Proof

First, observe that $V_{g}f(z+t\vec {\ell }(\beta ))$ is real analytic if and only if

$$\begin{aligned} V_{g}f\big (R( \beta )R( -\beta )(z+t\vec {\ell }(\beta ))\big )=V_{U( \beta )g}(U(\beta )f)\big (R(-\beta )z+t\vec {e}_1\big ) \end{aligned}$$

is real analytic. For $\beta \in {\mathbb {T}}$ one has that $U(\beta )g\in {\mathcal {M}}({\mathbb {R}})$ by Lemma 4 and $|U(\beta )g(t)|\lesssim e^{-\alpha |t|}$. Since the metaplectic rotation leaves $M^p_{1/\vartheta _s}({\mathbb {R}})$ invariant, it follows by [6, Corollary 26] that $V_{U( \beta )g}(U(\beta )f)\big (R(-\beta )z+t\vec {e}_1\big )$ is real analytic. $\square$

#### Proof of Theorem 5

First, observe that $g\in {\mathcal {M}}({\mathbb {R}})$ by Lemma 4. We may therefore apply Theorem 3. By Theorem 4, the set of weak limits of translates consists of finite shifts of $\varGamma$ and the sets of parallel lines and edges. As $V_gf(z-w)=e^{2\pi i \xi y}V_g\pi (w)f(z)$, and $M_{1/\vartheta _s}^p({\mathbb {R}})$ is invariant under time-frequency shifts, it follows that $z+\varGamma$ is a uniqueness set for $M_{1/\vartheta _s}^p({\mathbb {R}})$ if and only if $\varGamma$ is a uniqueness set for $M_{1/\vartheta _s}^p({\mathbb {R}})$.

We now show that every set of parallel lines (resp. parallel edges) is a uniqueness set. To show the two cases at once, we prove that any collection of parallel half lines $\{\eta k\vec {d}_1 +t\vec {d}_2:\ t\ge 0, k\in {\mathbb {Z}}\}$, $\vec {d_1},\vec {d}_2$ not colinear, and $\eta >0$, is a uniqueness set for $M_{1/\vartheta _s}^p({\mathbb {R}})$. Since $V_gf$ restricted to a line in ${\mathbb {R}}^2$ is real analytic by Lemma 5, it follows that $V_g f|_{\{\eta k\vec {d}_1 +t\vec {d}_2:\ t\ge 0, \ k\in {\mathbb {Z}}\}}=0$ implies $V_g f|_{\{\eta k\vec {d}_1 +t\vec {d}_2:\ t\in {\mathbb {R}}, \ k\in {\mathbb {Z}}\}}=0$.

The function $U(\theta )g$ is also a linear combination of Hermite functions and thus has only finitely many zeros. Therefore, it follows that $\bigcup _{k\in {\mathbb {Z}}}\widetilde{\text {supp}}(T_{\delta k}U(\theta )g)={\mathbb {R}}$ for every $\delta >0$. Finally, as $g\in A_{\vartheta _s}({\mathbb {R}})$, we may apply Proposition 3 to see that $V_g f|_{\{\eta k\vec {d}_1 +t\vec {d}_2:\ t\in {\mathbb {R}}, \ k\in {\mathbb {Z}}\}}=0$ implies $f=0$. $\square$

#### Corollary 2

Let $g\in \text {span}\{h_n: n\in {\mathbb {N}}_0\}$, $1\le p<\infty$, and $\eta >0$. Moreover, let $P\subset {\mathbb {R}}^2$ and $\{z_1,...,z_n\}\subset {\mathbb {R}}^2$ be such that the assumptions $(a)-(b)$ in Theorem 1 are satisfied. The collection of star shaped polygons $P_\eta$ and the path ${\mathcal {S}}(z_1,...,z_N)$ are Gabor sampling trajectories for $M^p({\mathbb {R}})$.

#### Proof

Take a line $\ell \subset {\mathbb {R}}^2$ that contains one of the edges of $P_\eta$ (resp. that contains the line segment $s(z_1,z_2)$) and consider the collection $\{\eta k\ell \}_{k\in {\mathbb {N}}}$. If $V_gf|_{P_\eta }=0$ (resp. $V_gf|_{{\mathcal {S}}(z_1,...,z_n)}=0$), then, as $V_gf$ is real analytic on any line $\eta k\ell$ and zero on a subset of nonzero measure, it follows that $V_gf|_{\{\eta k\ell \}_{k\in {\mathbb {N}}}}=0$. Arguing as before, we get $\bigcup _{k\in {\mathbb {N}}} \widetilde{\text {supp}}(T_{k\eta }U(\beta )g)={\mathbb {R}}$. Therefore, it follows that that $f=0$. $\square$

#### Corollary 3

Let $g\in \text {span}\{h_n: n\in {\mathbb {N}}_0\}$, $\eta >0$, and $1\le p<\infty$. The collection of concentric circles $O_\eta$ is a Gabor sampling trajectory for $M^p({\mathbb {R}})$.

#### Proof

For $g=\sum _{k=0}^n\alpha _k h_k$, we denote by *F* the polyanalytic function of order *n* given by $F:=\sum _{k=0}^n\alpha _k F_k$, where $F_k(z)=V_{h_k}f({\bar{z}})e^{\pi (z^2-{\bar{z}}^2)/4}e^{\pi |z|^2/2}$, see (6). Then $V_g f({\overline{z}})=0$ exactly when $F(z)=0$. Multiplying *F* by $z^n$ changes the zero set of *F* at most in the origin, and results in a reduced polyanalytic function. By assumption, and since $\overline{O_\eta }=O_\eta$ we hence have that $z^nF|_{O_\eta }=0$. By Lemma 1, it thus follows that $F(z)=0$ for every $z\in B_{R_0}(0)$, where $R_0$ can be chosen arbitrarily large. Consequently, $F(z)=0$ for every $z\in {\mathbb {C}}$ which implies that $f=0$ and $O_\eta$ is a uniqueness set for $M^p_{1/\vartheta _s}({\mathbb {R}})$. $\square$
